# Supplementary material for: Within-host evolution of Helicobacter pylori shaped by niche-specific adaptation, intragastric migrations and selective sweeps
Source: Nat Commun. 2019 May 22;10:2273. doi: 10.1038/s41467-019-10050-1 (PMC6531487; doi:10.1038/s41467-019-10050-1)
Supplement: Supplementary file 1 — Supplementary Information [file 41467_2019_10050_MOESM1_ESM.pdf]

Supplementary Information

Within-host evolution of *Helicobacter pylori* shaped by niche-specific adaptation, intragastric migrations and selective sweeps

Ailloud et al.

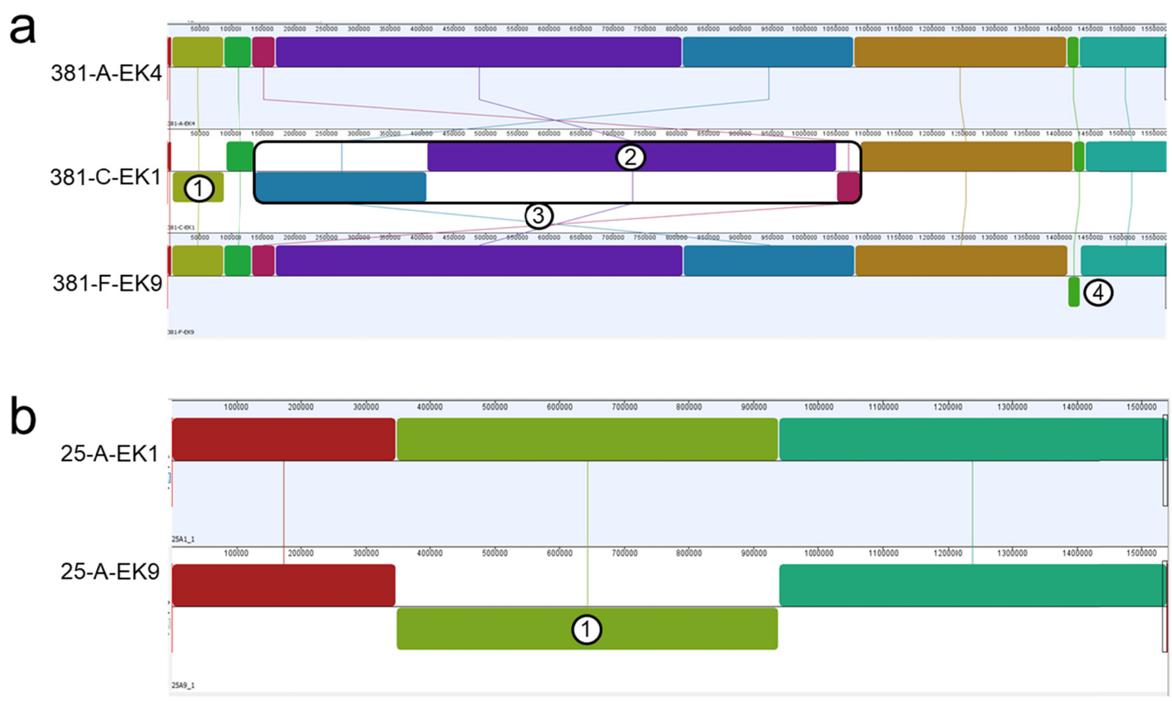

Supplementary Figure 1. a. Alignment of 3 SMRT genomes from patient 381. Reference genomes from patient 381 were aligned using progressiveMauve with default parameters. Blocks of similar colors represent homologous regions. The top and bottom section for each isolate in the alignment correspond to the positive and negative strands, respectively. Several genomic rearrangements were observed in patient 381: 1) and 2) recombination between mini-IS605 copies; 3) recombination between 23S copies; 4) recombination between *tlpA* and *tlpC*. **b**. Alignment of 2 SMRT genomes from patient 25. Reference genomes from patient 25 were aligned using progressiveMauve with default parameters. Blocks of similar colors represent homologous regions. The top and bottom section for each isolate in the alignment correspond to the positive and negative strands, respectively. A single genomic rearrangement was observed in patient 25: 1) recombination between HP0488-HP1116 paralogs.

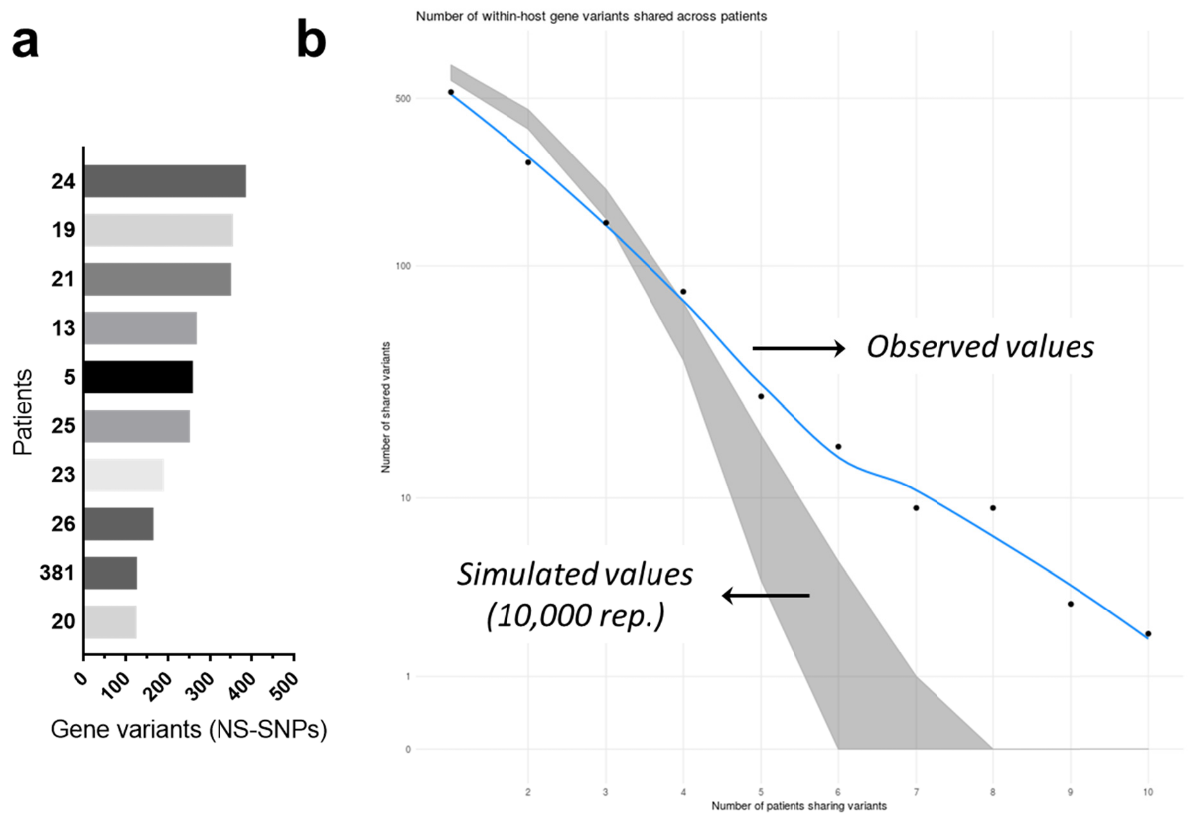

Supplementary Figure 2. Simulation of the expected frequency of shared within-host protein variants between multiple hosts. a. Number of genes containing non-synonymous mutations in each *H. pylori* population from the subset of 10 patients including fundus isolates. **b.** Simulated versus observed values of shared pools of polymorphic genes. Simulated values were obtained by random successive sampling according to the total pool of polymorphic genes observed in each population, 95% confidence intervals of the simulated values are shown as the grey area on the plot. Observed values are shown as dots.

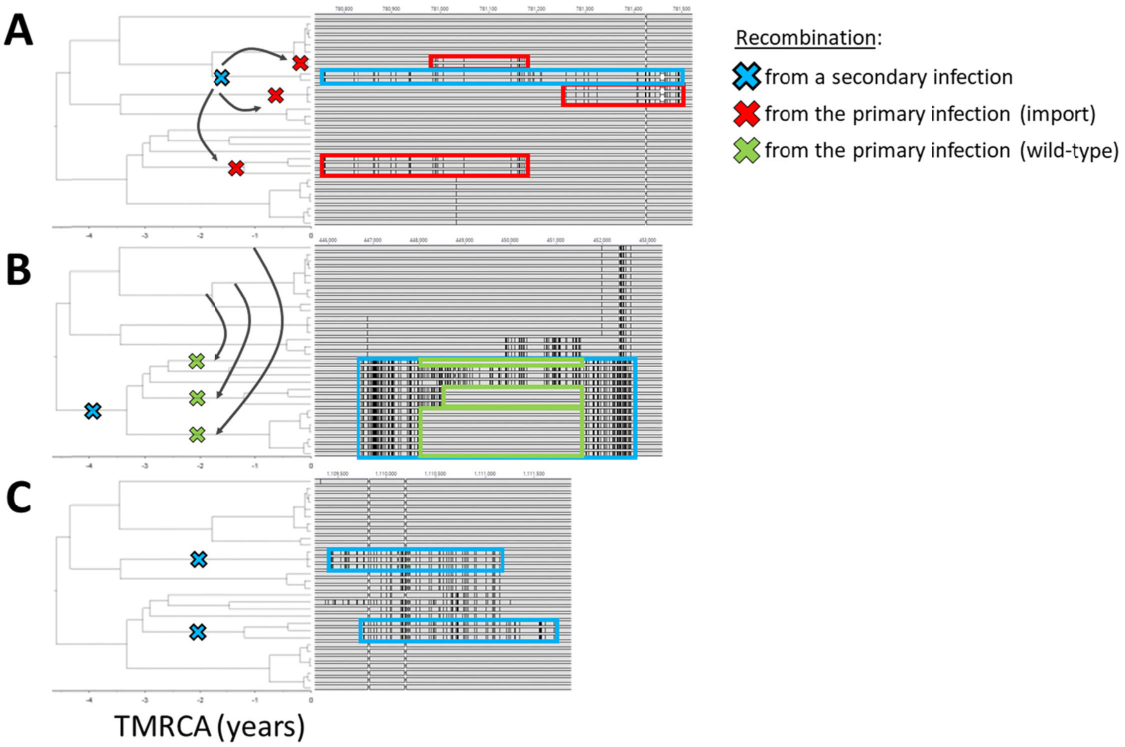

Supplementary Figure 3. Model for the intra-population distribution of imports within *H. pylori*. a. Single recombination event from the secondary infection followed by multiple recombination events from the recombined – primary infection – lineage. **b.** Single recombination event from the secondary infection followed by multiple recombination events from wild-type – primary infection – lineages. **c.** Multiple events from the secondary infection. These imports are usually not 100% similar suggesting they come from distinct lineages.
