# Supplementary material for: Within-host evolution of Helicobacter pylori shaped by niche-specific adaptation, intragastric migrations and selective sweeps
Source: Nat Commun. 2019 May 22;10:2273. doi: 10.1038/s41467-019-10050-1 (PMC6531487; doi:10.1038/s41467-019-10050-1)
Supplement: Supplementary file 3 — Reporting Summary [file 41467_2019_10050_MOESM3_ESM.pdf]

nature research

Corresponding author(s): *Double-blind peer review submissions: write DBPR and your manuscript number here instead of author names.*

Last updated by author(s): *YYYY-MM-DD*

Reporting Summary

Nature Research wishes to improve the reproducibility of the work that we publish. This form provides structure for consistency and transparency in reporting. For further information on Nature Research policies, see Authors & Referees and the Editorial Policy Checklist.

Statistics

For all statistical analyses, confirm that the following items are present in the figure legend, table legend, main text, or Methods section.

n/a	Confirmed	
☐	☒	The exact sample size (*n*) for each experimental group/condition, given as a discrete number and unit of measurement
☐	☒	A statement on whether measurements were taken from distinct samples or whether the same sample was measured repeatedly
☐	☒	The statistical test(s) used AND whether they are one- or two-sided<br>*Only common tests should be described solely by name; describe more complex techniques in the Methods section.*
☒	☐	A description of all covariates tested
☐	☒	A description of any assumptions or corrections, such as tests of normality and adjustment for multiple comparisons
☐	☒	A full description of the statistical parameters including central tendency (e.g. means) or other basic estimates (e.g. regression coefficient) AND variation (e.g. standard deviation) or associated estimates of uncertainty (e.g. confidence intervals)
☐	☒	For null hypothesis testing, the test statistic (e.g. *F*, *t*, *r*) with confidence intervals, effect sizes, degrees of freedom and *P* value noted<br>*Give P values as exact values whenever suitable.*
☒	☐	For Bayesian analysis, information on the choice of priors and Markov chain Monte Carlo settings
☒	☐	For hierarchical and complex designs, identification of the appropriate level for tests and full reporting of outcomes
☒	☐	Estimates of effect sizes (e.g. Cohen's *d*, Pearson's *r*), indicating how they were calculated

Our web collection on statistics for biologists contains articles on many of the points above.

Software and code

Policy information about availability of computer code

Data collection	Illumina paired-end reads were assembled using SPAdes 3.9.0. Pacbio RSII reads were assembled using HGAP3. Genomes were annotated with Prokka 1.7, Infernal and Rfam 11.0
Data analysis	Genome multiple alignments were created with BWA-MEM 0.7.12. Phylogenetic trees were generated with RAxML 8.2.9 or FastTree. Recombination was analyzed with ClonalFrameML 1.0. Character transition rates (i.e. migration of strains between stomach parts) was analyzed with the ape 4.1, geiger 2.0.6 and phytools 0.6 R packages.

For manuscripts utilizing custom algorithms or software that are central to the research but not yet described in published literature, software must be made available to editors/reviewers. We strongly encourage code deposition in a community repository (e.g. GitHub). See the Nature Research guidelines for submitting code & software for further information.

Data

Policy information about availability of data

All manuscripts must include a data availability statement. This statement should provide the following information, where applicable:

- Accession codes, unique identifiers, or web links for publicly available datasets
- A list of figures that have associated raw data
- A description of any restrictions on data availability

Reference genomes and raw sequencing reads are available on the NCBI database (BioProject PRJNA490474). GFF annotation files are available in the FigShare repository (https://figshare.com/s/c84f82df296b88dd75ee)

Field-specific reporting

Please select the one below that is the best fit for your research. If you are not sure, read the appropriate sections before making your selection.

☒ Life sciences ☐ Behavioural & social sciences ☐ Ecological, evolutionary & environmental sciences

For a reference copy of the document with all sections, see nature.com/documents/nr-reporting-summary-flat.pdf

Life sciences study design

All studies must disclose on these points even when the disclosure is negative.

Sample size	No sample size calculation was performed.
Data exclusions	No data were excluded from the analysis.
Replication	Reproducibility of the experimental findings was ensured by conducting analysis on a set of n=10 patients.
Randomization	Allocation was random.
Blinding	Investigators were blinded during data collection but not during analysis.

Reporting for specific materials, systems and methods

We require information from authors about some types of materials, experimental systems and methods used in many studies. Here, indicate whether each material, system or method listed is relevant to your study. If you are not sure if a list item applies to your research, read the appropriate section before selecting a response.

Materials & experimental systems

n/a	Involved in the study	
☒	☐	Antibodies
☒	☐	Eukaryotic cell lines
☒	☐	Palaeontology
☒	☐	Animals and other organisms
☐	☒	Human research participants
☒	☐	Clinical data

Methods

n/a	Involved in the study	
☒	☐	ChIP-seq
☒	☐	Flow cytometry
☒	☐	MRI-based neuroimaging

Human research participants

Policy information about studies involving human research participants

Population characteristics

Patient 169: 50 years old female with chronic gastritis and no prior Helicobacter pylori eradication treatment
Patient 173: 44 years old female with chronic gastritis and prior Helicobacter pylori eradication treatment
Patient 478: 70 years old male with atrophic gastritis/intestinal metaplasia and prior Helicobacter pylori eradication treatment
Patient 280: 76 years old female with chronic gastritis and prior Helicobacter pylori eradication treatment
Patient 476: 34 years old male with chronic gastritis and no prior Helicobacter pylori eradication treatment
Patient 479: 69 years old male with atrophic gastritis and no prior Helicobacter pylori eradication treatment
Patient 21: 58 years old male with chronic gastritis and no prior Helicobacter pylori eradication treatment
Patient 381: 42 years old female with atrophic gastritis and prior Helicobacter pylori eradication treatment
Patient 5: 44 years old female with chronic gastritis and prior Helicobacter pylori eradication treatment
Patient 26: 47 years old female with atrophic gastritis and unkown Helicobacter pylori eradication treatment
Patient 23: 30 years old male with chronic gastritis and no prior Helicobacter pylori eradication treatment
Patient 13: 54 years old female with chronic gastritis and no prior Helicobacter pylori eradication treatment
Patient 20: 55 years old female with chronic gastritis and prior Helicobacter pylori eradication treatment
Patient 24: 33 years old male with chronic gastritis and no prior Helicobacter pylori eradication treatment
Patient 25: 56 years old male with chronic gastritis and unknown Helicobacter pylori eradication treatment
Patient 19: 32 years old female with chronic gastritis and no prior Helicobacter pylori eradication treatment

Recruitment

Participants were recruited on the basis of being positive for Helicobacter pylori infection based on clinical symptoms and UBT (urea breath test) kit results.

Ethics oversight

Ethics committee at the Otto-von-Guericke University, Magdeburg, Germany (protocol number 80/11)

Note that full information on the approval of the study protocol must also be provided in the manuscript.
