# Supplementary material for: Within-host evolution of Helicobacter pylori shaped by niche-specific adaptation, intragastric migrations and selective sweeps
Source: Nat Commun. 2019 May 22;10:2273. doi: 10.1038/s41467-019-10050-1 (PMC6531487; doi:10.1038/s41467-019-10050-1)
Supplement: Supplementary file 4 — Description of Additional Supplementary Files [file 41467_2019_10050_MOESM4_ESM.pdf]

Description of Additional Supplementary Files

File Name: Supplementary Data 1
Description: Genome characteristics of all sequenced *H. pylori* isolates. Assembly metrics of draft and complete genomes.

File Name: Supplementary Data 2
Description: High-frequency host-variable genes within 10 *H. pylori* populations. Functionally annotated version of the data presented in Table 2.

File Name: Supplementary Data 3
Description: Non-synonymous mutations associated with gastric regions in 10 *H. pylori* populations. Genes containing polymorphisms with significant association to gastric regions. FDR-adjusted p-values are displayed for Fisher's exact test performed with the original sampling region of each isolate (uncorrected), the region corrected for recent migration event up to 1 year or 6 months.
